# Supplementary material for: Sound-meaning associations allow listeners to infer the meaning of foreign language words
Source: Commun Psychol. Author manuscript; Available in PMC 2023 Dec 27. (PMC10751683; doi:10.1038/s44271-023-00030-z)
Supplement: Supplementary Material [file NIHMS1948110-supplement-Supplementary_Material.docx]

**Supplementary Information**

**Table S1.** Tukey-adjusted pairwise comparisons of phonetic distance from English in each part of speech by language group for native English speakers

| Language Group | Contrast | Estimate | SE | 95% CI | df | t | p |  |
| --- | --- | --- | --- | --- | --- | --- | --- | --- |
| Japonic-Sino-Tai | Adjective - Noun | -0.05 | 0.04 | [-0.13, 0.04] | 115 | -1.34 | 0.376 |  |
|  | Adjective - Verb | 0.03 | 0.04 | [-0.06, 0.11] | 115 | 0.75 | 0.750 |  |
|  | Noun - Verb | 0.07 | 0.04 | [-0.01, 0.16] | 115 | 2.06 | 0.102 |  |
| Slavic | Adjective - Noun | 0.06 | 0.04 | [-0.03, 0.14] | 115 | 1.59 | 0.253 |  |
|  | Adjective - Verb | 0.05 | 0.04 | [-0.03, 0.14] | 115 | 1.45 | 0.318 |  |
|  | Noun - Verb | -0.01 | 0.04 | [-0.09, 0.08] | 115 | -0.14 | 0.989 |  |
| Romance | Adjective - Noun | 0.21 | 0.04 | [0.13, 0.30] | 115 | 5.96 | <.0001 | *** |
|  | Adjective - Verb | 0.09 | 0.04 | [0.01, 0.18] | 115 | 2.62 | 0.027 | * |
|  | Noun - Verb | -0.12 | 0.04 | [-0.20, -0.03] | 115 | -3.35 | 0.003 | ** |

**Table S2.** Tukey-adjusted effects of part of speech on accuracy by language group and form-meaning regularity for native English speakers

| Form-Meaning Regularity | Language Group | Contrast | Odds Ratio | z | 95% CI | p |  |
| --- | --- | --- | --- | --- | --- | --- | --- |
| Less Regularity | Japonic-Sino-Tai | Adjective / Noun | 1.11 | 0.66 | [0.77, 1.58] | 0.079 |  |
|  |  | Adjective / Verb | 0.91 | -0.62 | [0.64, 1.30] | 0.081 |  |
|  |  | Noun / Verb | 0.82 | -1.30 | [0.58, 1.17] | 0.396 |  |
|  | Romance | Adjective / Noun | 0.31 | -6.19 | [0.20, 0.49] | < 0.001 | *** |
|  |  | Adjective / Verb | 0.98 | -0.10 | [0.66, 1.47] | 0.994 |  |
|  |  | Noun / Verb | 3.14 | 6.08 | [2.02, 4.88] | < 0.001 | *** |
|  | Slavic | Adjective / Noun | 1.07 | 0.38 | [0.72, 1.59] | 0.924 |  |
|  |  | Adjective / Verb | 0.84 | -0.96 | [0.55, 1.28] | 0.605 |  |
|  |  | Noun / Verb | 0.79 | -1.43 | [0.54, 1.16] | 0.326 |  |
| Greater Regularity | Japonic-Sino-Tai | Adjective / Noun | 1.05 | 0.31 | [0.72, 1.54] | 0.947 |  |
|  |  | Adjective / Verb | 1.11 | 0.64 | [0.77, 1.60] | 0.796 |  |
|  |  | Noun / Verb | 1.05 | 0.32 | [0.72, 1.53] | 0.946 |  |
|  | Romance | Adjective / Noun | 0.65 | -2.42 | [0.43, 0.99] | 0.041 | * |
|  |  | Adjective / Verb | 0.49 | -4.27 | [0.33, 0.72] | < 0.001 | *** |
|  |  | Noun / Verb | 0.74 | -1.60 | [0.48, 1.15] | 0.244 |  |
|  | Slavic | Adjective / Noun | 0.64 | -2.71 | [0.44, 0.94] | 0.018 | * |
|  |  | Adjective / Verb | 0.72 | -2.05 | [0.50, 1.05] | 0.100 |  |
|  |  | Noun / Verb | 1.13 | 0.71 | [0.76, 1.68] | 0.760 |  |

**Table S3.** Tukey-adjusted effects of part of speech on accuracy by language group and form-meaning regularity for native Spanish speakers

| Form-Meaning Regularity | Language | Contrast | Odds Ratio | z | 95% CI | p |  |
| --- | --- | --- | --- | --- | --- | --- | --- |
| Less Regularity | English | Adjective / Noun | 0.38 | -2.82 | [0.17, 0.85] | 0.013 | * |
|  |  | Adjective / Verb | 0.84 | -0.65 | [0.44, 1.60] | 0.792 |  |
|  |  | Noun / Verb | 2.19 | 2.15 | [0.93, 5.13] | 0.080 |  |
|  | Japanese | Adjective / Noun | 1.24 | 0.88 | [0.70, 2.23] | 0.652 |  |
|  |  | Adjective / Verb | 0.67 | -1.49 | [0.36, 1.26] | 0.294 |  |
|  |  | Noun / Verb | 0.54 | -2.38 | [0.29, 0.99] | 0.045 | * |
|  | Polish | Adjective / Noun | 0.78 | -0.87 | [0.40, 1.52] | 0.659 |  |
|  |  | Adjective / Verb | 0.57 | -2.14 | [0.31, 1.06] | 0.082 |  |
|  |  | Noun / Verb | 0.73 | -1.12 | [0.38, 1.41] | 0.500 |  |
| Greater Regularity | English | Adjective / Noun | 0.36 | -3.42 | [0.18, 0.72] | 0.002 | ** |
|  |  | Adjective / Verb | 0.55 | -2.07 | [0.28, 1.08] | 0.096 |  |
|  |  | Noun / Verb | 1.52 | 1.62 | [0.83, 2.78] | 0.239 |  |
|  | Japanese | Adjective / Noun | 0.71 | -1.28 | [0.37, 1.33] | 0.404 |  |
|  |  | Adjective / Verb | 0.87 | -0.55 | [0.47, 1.59] | 0.845 |  |
|  |  | Noun / Verb | 1.23 | 0.77 | [0.66, 2.30] | 0.722 |  |
|  | Polish | Adjective / Noun | 1.32 | 0.84 | [0.61, 2.89] | 0.680 |  |
|  |  | Adjective / Verb | 2.69 | 3.41 | [1.36, 5.33] | 0.002 | ** |
|  |  | Noun / Verb | 2.04 | 2.25 | [0.97, 4.28] | 0.063 |  |
