# Supplementary material for: Sound-meaning associations allow listeners to infer the meaning of foreign language words
Source: Commun Psychol. 2023 Nov 2;1:30. doi: 10.1038/s44271-023-00030-z (PMC10751683; doi:10.1038/s44271-023-00030-z)
Supplement: Supplementary file 2 [file 44271_2023_30_MOESM3_ESM.pdf]

nature portfolio

Corresponding author(s): Hayakawa, Sayuri

Last updated by author(s): 9/20/2023

Reporting Summary

Nature Portfolio wishes to improve the reproducibility of the work that we publish. This form provides structure for consistency and transparency in reporting. For further information on Nature Portfolio policies, see our Editorial Policies and the Editorial Policy Checklist.

Statistics

For all statistical analyses, confirm that the following items are present in the figure legend, table legend, main text, or Methods section.

n/a	Confirmed	
☐	☒	The exact sample size (*n*) for each experimental group/condition, given as a discrete number and unit of measurement
☐	☒	A statement on whether measurements were taken from distinct samples or whether the same sample was measured repeatedly
☐	☒	The statistical test(s) used AND whether they are one- or two-sided<br>*Only common tests should be described solely by name; describe more complex techniques in the Methods section.*
☐	☒	A description of all covariates tested
☐	☒	A description of any assumptions or corrections, such as tests of normality and adjustment for multiple comparisons
☐	☒	A full description of the statistical parameters including central tendency (e.g. means) or other basic estimates (e.g. regression coefficient) AND variation (e.g. standard deviation) or associated estimates of uncertainty (e.g. confidence intervals)
☐	☒	For null hypothesis testing, the test statistic (e.g. *F*, *t*, *r*) with confidence intervals, effect sizes, degrees of freedom and *P* value noted<br>*Give P values as exact values whenever suitable.*
☒	☐	For Bayesian analysis, information on the choice of priors and Markov chain Monte Carlo settings
☐	☒	For hierarchical and complex designs, identification of the appropriate level for tests and full reporting of outcomes
☐	☒	Estimates of effect sizes (e.g. Cohen's *d*, Pearson's *r*), indicating how they were calculated

Our web collection on statistics for biologists contains articles on many of the points above.

Software and code

Policy information about availability of computer code

Data collection	Data were collected online using the Qualtrics platform.
Data analysis	The data were analyzed using the LingPy Python library, as well as lme4, lmerTest, and emmeans packages in R (version 4.1.2).

For manuscripts utilizing custom algorithms or software that are central to the research but not yet described in published literature, software must be made available to editors and reviewers. We strongly encourage code deposition in a community repository (e.g. GitHub). See the Nature Portfolio guidelines for submitting code & software for further information.

Data

Policy information about availability of data

All manuscripts must include a data availability statement. This statement should provide the following information, where applicable:

- Accession codes, unique identifiers, or web links for publicly available datasets
- A description of any restrictions on data availability
- For clinical datasets or third party data, please ensure that the statement adheres to our policy

The data that support the findings of this study can be accessed at https://osf.io/4ez8v/

Research involving human participants, their data, or biological material

Policy information about studies with human participants or human data. See also policy information about sex, gender (identity/presentation), and sexual orientation and race, ethnicity and racism.

Reporting on sex and gender	Participants were recruited online via the Prolific recruitment platform, where we specified that the distribution of genders should be as balanced as possible. Following pre-screening, the final sample for Experiment 1 included 62 self-reported females and 72 self-reported males, and the final sample for Experiment 2 included 17 self-reported females and 29 self-reported males. Gender was not included in the analyses as gender effects were beyond the scope of the present investigation.
Reporting on race, ethnicity, or other socially relevant groupings	Participants' language background and experience was used to determine eligibility in the two studies. Inclusion criteria included self-reported monolingual, English native language status for Experiment 1 and monolingual, Spanish native language status for Experiment 2. Participants were asked to indicate their "native language", identify any other languages they had "experience" with, and to rate their "proficiency" on a 0-10 scale in a series of languages relevant to the present investigation (English, Spanish, French, Romanian, Polish, Ukrainian, Russian, Japanese, Mandarin, Thai). The majority of participants in Experiment 1 self-identified as non-Hispanic White (77.6%), followed by Black or African American (13.4%), More than One Race (5.2%), and Asian (2.3%). An additional two participants (1.5%) declined to answer. The majority of participants in Experiment 2 self-identified as Hispanic (78.3%), followed by non-Hispanic White (10.9%), More than One Race (8.7%), and Native American (2.1%).
Population characteristics	In addition to language background, race/ethnicity, and gender (see above), participants reported their current age. Mean ages at the time of the experiment were 35.61 years old (SD = 11.60) and 28.63 years old (SD = 7.03) in Experiments 1 and 2, respectively.
Recruitment	Participants were recruited online via the Prolific recruitment platform. They were first screened for eligibility based on information participants had previously submitted to Prolific (which included monolingual, native language status and fluency in English for Experiment 1, and in Spanish for Experiment 2). Eligible participants then completed a second pre-screening via the Qualtrics platform where they indicated their level of proficiency in a series of languages relevant to the investigation (see above).
Ethics oversight	Northwestern University Institutional Review Board

Note that full information on the approval of the study protocol must also be provided in the manuscript.

Field-specific reporting

Please select the one below that is the best fit for your research. If you are not sure, read the appropriate sections before making your selection.

☐ Life sciences ☒ Behavioural & social sciences ☐ Ecological, evolutionary & environmental sciences

For a reference copy of the document with all sections, see nature.com/documents/nr-reporting-summary-flat.pdf

Behavioural & social sciences study design

All studies must disclose on these points even when the disclosure is negative.

Study description	The two reported studies utilized quantitative experimental methods.
Research sample	Participants for Experiment 1 included 134 monolingual, native English speakers (46% female) with a mean age of 35.61 years old (SD = 11.60) residing in the United States. Participants for Experiment 2 included 46 monolingual, native Spanish speakers (37% female) with a mean age of 28.63 years old (SD = 7.03) residing outside of the United States. Both samples were recruited online using the Prolific platform in order to access a broad and representative sample from each population.
Sampling strategy	Participants were prescreened for eligibility via Prolific based on previously reported demographic and language background information. All eligible participants then had the opportunity to view the study description and complete a secondary screening assessing more detailed language background information if they were interested in participating. An a priori power analysis for logistic regression using G*Power 3.1.9.71 with an alpha level of 0.05 and estimated power of 0.80 indicated a necessary sample size of 98 to detect an odds ratio of 1.86 (H0 = 0.5 and H1 = 0.65 based on pilot data).
Data collection	All data were collected online via the Qualtrics platform. Participants therefore completed the experiment on their own without an experimenter present. Data included typed responses, selected choices, and response times (no audio or video was collected).
Timing	Data for Experiment 1 were collected between 2022-02-22 and 2022-03-18. Data for Experiment 2 were collected between 2023-08-01 and 2023-08-13.
Data exclusions	Two participants were excluded from Experiment 1 due to technical issues resulting in incomplete recording of the data. One participant was excluded from Experiment 2 due to perfect performance on the English word-pair task despite reporting minimal English experience (which was a pre-determined inclusion criterion).

Non-participation	A total of 18 participants dropped out of Experiment 1 after passing eligibility screening and giving consent. No reason was given. No participants dropped out of Experiment 2.
Randomization	Participants in Experiment 1 were randomly assigned to three of nine possible language conditions using Qualtrics' built-in algorithm. Participants in Experiment 2 all completed the same three language conditions (i.e. no random assignment to condition). In both experiments, the order of language blocks and order of trials within blocks were randomized for each participant using Qualtrics' built-in algorithms.

Reporting for specific materials, systems and methods

We require information from authors about some types of materials, experimental systems and methods used in many studies. Here, indicate whether each material, system or method listed is relevant to your study. If you are not sure if a list item applies to your research, read the appropriate section before selecting a response.

Materials & experimental systems

n/a	Involved in the study
☒	☐ Antibodies
☒	☐ Eukaryotic cell lines
☒	☐ Palaeontology and archaeology
☒	☐ Animals and other organisms
☒	☐ Clinical data
☒	☐ Dual use research of concern
☒	☐ Plants

Methods

n/a	Involved in the study
☒	☐ ChIP-seq
☒	☐ Flow cytometry
☒	☐ MRI-based neuroimaging
